# AI-Based EMG Reporting: A Randomized Controlled Trial

**DOI:** 10.1007/s00415-025-13261-3

**Published:** 2025-08-22

**Authors:** Alon Gorenshtein, Yana Weisblat, Mohamed Khateb, Gilad Kenan, Irina Tsirkin, Galina Fayn, Semion Geller, Shahar Shelly

**Affiliations:** 1 Department of Neurology, Rambam Health Care Campus, Haifa, Israel; 2 Azrieli Faculty of Medicine, Bar-Ilan University, Safed, Israel; 3 AI Neurology Laboratory, Ruth and Bruce Rapaport Faculty of Medicine, Technion Institute of Technology, 3525408 Haifa, Israel; 4 Department of Neurology, University Health Network, University of Toronto, Toronto, ON Canada; 5 Sackler Faculty of Medicine, Tel Aviv University, Tel Aviv, Israel; 6 Department of Neurology, Shamir Medical Center, Tzrifin, Israel; 7 Department of Neurology, Assuta Medical Center, Ashdod, Israel; 8 Department of Neurology, Mayo Clinic, Rochester, MN US

**Keywords:** Large language models, Neurology, Electrodiagnostic study, Neuromuscular, Multi-AI agents, Artificial intelligence, Nerve conduction study, Electromyogram, Randomized controlled trial

## Abstract

**Background and objectives:**

Accurate interpretation of electrodiagnostic (EDX) studies is essential for the diagnosis and management of neuromuscular disorders. Artificial intelligence (AI) based tools may improve consistency and quality of EDX reporting and reduce workload. The aim of this study is to evaluate the performance of an AI‐assisted, multi‐agent framework (INSPIRE) in comparison with standard physician interpretation in a randomized controlled trial (RCT).

**Methods:**

We prospectively enrolled 200 patients (out of 363 assessed for eligibility) referred for EDX. Patients were randomly assigned to either a control group (physician‐only interpretation) or an intervention group (physician–AI interpretation). Three board‐certified physicians, rotated across both arms. In the intervention group, an AI‐generated preliminary report was combined with the physician’s independent findings (human–AI integration). The primary outcome was EDX report quality using score we developed named AI-Generated EMG Report Score (AIGERS; score range, 0–1, with higher scores indicating more accurate or complete reports). Secondary outcomes included a physician‐reported AI integration rating score (PAIR) and a compliance survey evaluating ease of AI adoption.

**Results:**

Of the 200 enrolled patients, 100 were allocated to AI‐assisted interpretation and 100 to physician‐only reporting. While AI‐generated preliminary reports offered moderate consistency on the AIGERS metric, the integrated (physician–AI) approach did not significantly outperform physician‐only methods. Despite some anecdotal advantages such as efficiency in suggesting standardized terminology quantitatively, the AIGERS scores for physician–AI integration was nearly the same as those in the physician‐only arm and did not reach statistical significance (*p* > 0.05 for all comparisons). Physicians reported variable acceptance of AI suggestions, expressing concerns about the interpretability of AI outputs and workflow interruptions. Physician–AI collaboration scores showed moderate trust in the AI’s suggestions (mean 3.7/5) but rated efficiency (2.0/5), ease of use (1.7/5), and workload reduction (1.7/5) as poor, indicating usability challenges and workflow interruptions.

**Discussion:**

In this single‐center, randomized trial, AI‐assisted EDX interpretation did not demonstrate a significant advantage over conventional physician‐only interpretation. Nevertheless, the AI framework may help reduce workload and documentation burdens by handling simpler, routine EDX tests freeing physicians to focus on more complex cases that require greater expertise.

**Trial registration:**

ClinicalTrials.gov Identifier: NCT06902675

**Supplementary Information:**

The online version contains supplementary material available at 10.1007/s00415-025-13261-3.

## Introduction

Artificial intelligence (AI) has demonstrated significant potential in clinical environments, particularly in diagnostic capabilities that may rival or even exceed those of human physicians.[1] Majority of studies to date have concentrated on AI's performance relative to physicians without providing a clear vision of the future integration of these technologies in clinical practice alongside specialists [2, 3]. This issue is exacerbated when we examine the assessment of cutting-edge AI, large language models (LLMs). LLMs (Chat-GPT, Gemini, etc.) are neural-network architectures trained on billions of words to predict the next token in a sequence. Through this simple objective they acquire an implicit knowledge base that can be queried with natural language, enabling step-by-step reasoning, text summarization and domain-specific question answering [4]. As of today much of the evaluation of LLM’s performance relies on multiple-choice questions of licensing exams, which, while useful for training physicians, do not adequately reflect the performance of physicians in actual clinical settings [2, 3, 5]. High test scores do not necessarily correlate with clinical efficacy for these models [6–8].

Instead, it is essential to perceive LLMs and AI as complementary tools designed to alleviate physician and non-physician workload, thereby enhancing patient care [9, 10]. The most effective assessment of these tools should occur through their direct implementation in clinical environments, allowing for real-world performance evaluation [11]. Recently AI studies have started to be conducted in the form of randomized controlled trials (RCT) [1, 12]. However, a significant limitation in existing studies is the lack of integration of physician factors in the clinical process [13, 14]. Some studies focus only on the theoretical possibility of AI–physician cooperation [13, 14] or relying on AI systems without humans in the loop [15]. Exploring how physicians’ expertise can refine AI tools may lead to solutions that surpass current physicians’ capabilities and reduce workload [16].

Neuromuscular electrodiagnostic testing (EDX), comprising electromyography (EMG) and nerve conduction studies (NCS), which are specialized procedures used to assess the electrical activity of muscles and nerves EDX represents fundamental diagnostic tools in neurology [17]. They serve as an extension of the clinical neurological examination [18], facilitating peripheral nerve disease monitoring and treatment response assessment [19]. Although their importance, EDX in the United States faces persistent challenges, with a significant proportion conducted by non-physicians. In 2004, it was reported that 16.9% of EDX encounters were performed by non-physician providers, who often evaluated younger patients with fewer comorbidities. [20]. In addition, EDX test are highly variable, we have previously showed various differences related to age and sex in a large cohort of patients [21]. These practical constraints make EDX an ideal testbed for evaluating AI-assisted, physician-supervised reporting. 

We conducted an RCT to evaluate the performance of physicians using the multi-AI-agents (INSPIRE) platform in generating EDX reports, compared to those who rely on conventional methods without AI assistance. In addition, the study aims to investigate physicians'readiness to engage with AI tools vs. their inclination toward traditional reporting methodologies. 

## Materials and methods

### Calibration phase

Before initiating the trial, we conducted test runs of the AI tool with voluntarily participating healthy individuals and retrospectively pathological EDX reports. This testing was essential for integrating the AI framework into our document formats, including tables, figures, and text. In addition, we assessed the optimal number of agents required, assigning each a specific task. After 2 weeks of testing, from January 5th, 2025, to January 19th, 2025, the AI framework was successfully adjusted to meet our clinic's needs. 

### Study groups and design

**Fig. 1:**
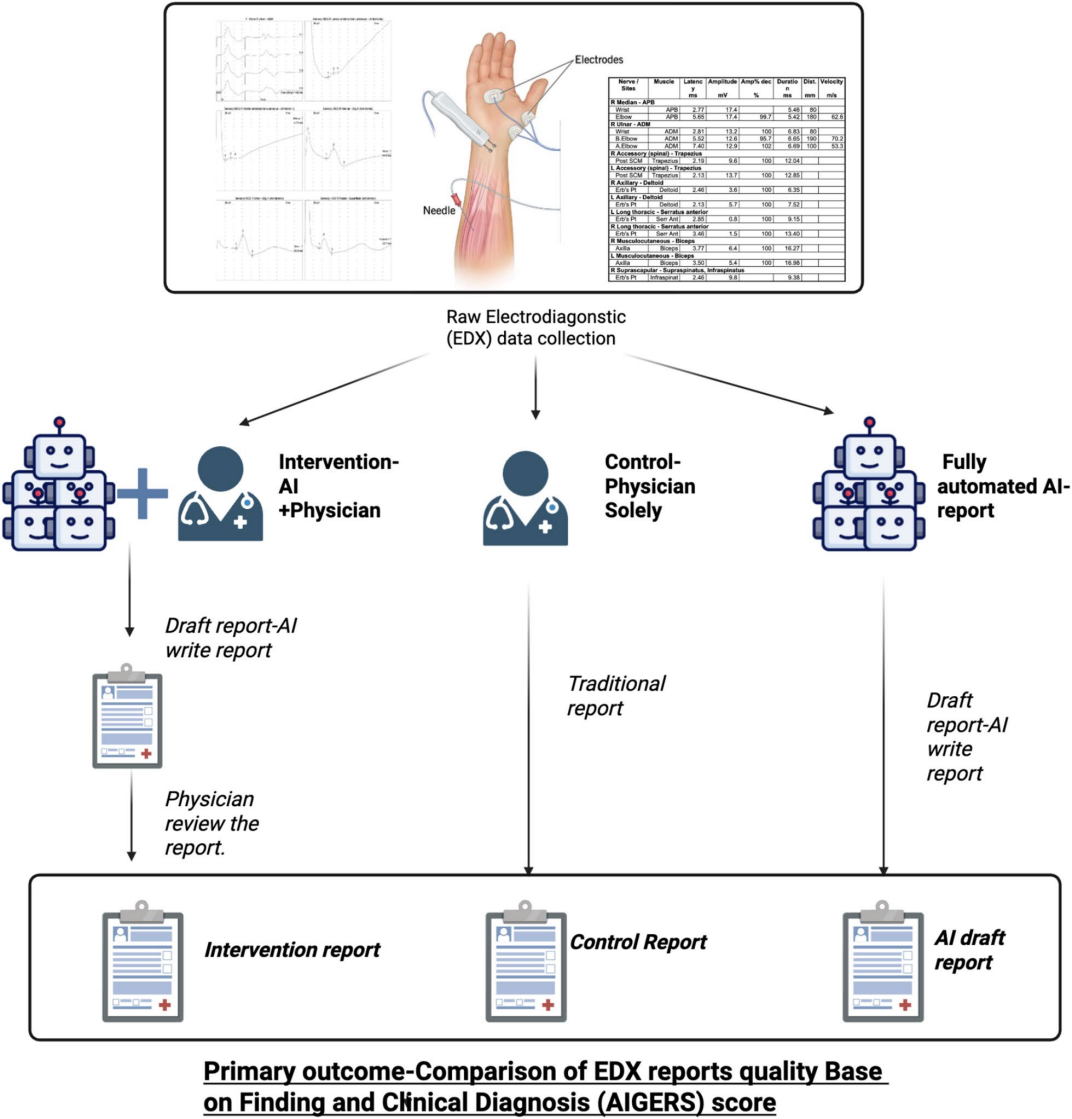
Schematic of the study arms and report generation process description. This diagram illustrates the three arms of the study for generating and evaluating raw electrodiagnostic (EDX) data comprising electromyography and nerve-conduction studies: (1) an AI-only approach that produces a fully automated report (part of the intervention), (2) a physician-only (control) approach using traditional methods, and (3) an AI + physician (intervention) approach in which the AI drafts a preliminary report (fully automated report) that is then reviewed and finalized by a physician. The primary outcome (AIGERS score) compares report quality among the three arms on the basis of both EDX findings and clinical diagnostic accuracy

The study was approved by our local IRB committee. We included all patients who underwent EDX tests at the outpatient EMG clinic between January 21, 2025, and February 21, 2025. We excluded patients who underwent a single fiber test or any incomplete EDX test (e.g., lacking EMG components), pediatric patients (age < 18), inpatient patients from the neurology department, and those who underwent EDX 3 months prior. The patients were randomly allocated to two groups, the AI intervention group (AI–physician report) and the control (physician report). To avoid observer bias, each day the physicians in the clinic would transfer to the other group. By the end of the study, we had three groups of reports: 1) AI–human reports: that group allowed first the AI to write the report and then was overseen and revised by the physician; 2) control: this was the standard physician report; and 3) AI: fully automated report generated. Figure 1 demonstrates the study design.

### Metrics

To assess the quality of the three groups’ reports, we developed two scores to optimize the evaluation of performance between all performers. We used a similar methodology we use in the educational process to evaluate EMG. We abbreviated this as the AIGERS SCORE meaning AI-Generated EMG Report Score (AIGERS). This scoring framework evaluates performance across two key criteria, emphasizing the model’s ability to detect findings and provide accurate diagnoses. Variables are; findings (F)—50% weight: scores range from 0 to 1, where a score of 0 indicates the model missed most findings in the original report, while a score of 1 signifies that all findings were accurately captured**.** Clinical diagnosis (CD)—50% weight**—**to evaluate the model’s ability to reach accurate medical diagnoses based on the findings. Scores range from −5–0, with critical errors heavily penalized (e.g., misclassifying a normal test as abnormal or vice versa results in a score of −5). Lesser errors, such as failing to localize a finding or including an additional mild incorrect diagnosis, incur smaller penalties (e.g., − 1). All scores were normalized to a range of 0–1 prior to calculating the AIGERS score, preserving the proportional impact of each component. Further details regarding AIGERS can be found in the supplementary material.

Evaluation of the Findings and Clinical Diagnosis scores involved a manual review of the three types of report (AI–physician, AI, and human). This review process was conducted by three senior neuromuscular specialists (M.K., G.K.M I.T), all three physicians were blinded to the group of the report and did not practice as part of the clinical trial itself, ensuring consistency and lack of bias. The study was considered normal if all AANEM NCS data were within normal limits apart from distal sensory responses in the sural nerve among individuals aged over 70 years. 

To evaluate the quality of physician–AI collaboration in AI-generated EDX reports we assessed through a survey containing four questions (Q1–Q4) (25% weight) each scored from 0 to 1. Q1, focused on Revisions, evaluated the amount of revision effort required from the physicians. Q2 dealt with Text Fixes, measuring how much editing was necessary to improve the report's wording. Q3, concerning Report Usability, looked at how practical and comprehensive the report was from the physicians'perspective. Finally, Q4 examined Trust in the AI report, gauging the confidence physicians had in the generated results. A higher score (closer to 1) indicates fewer modifications needed (Q1, Q2, Q3), fewer hallucinations (Q3, Q4), and a stronger alignment with physician standards (Q2, Q3). The detailed scoring rubric can be found in the Supplementary materials.

In addition, physicians took a survey at the end of the trial. These scores were correlated with the mean AIGERS and collaboration score of the reports the physician was tasked to cooperate with. 

### Intervention

**Fig. 2:**
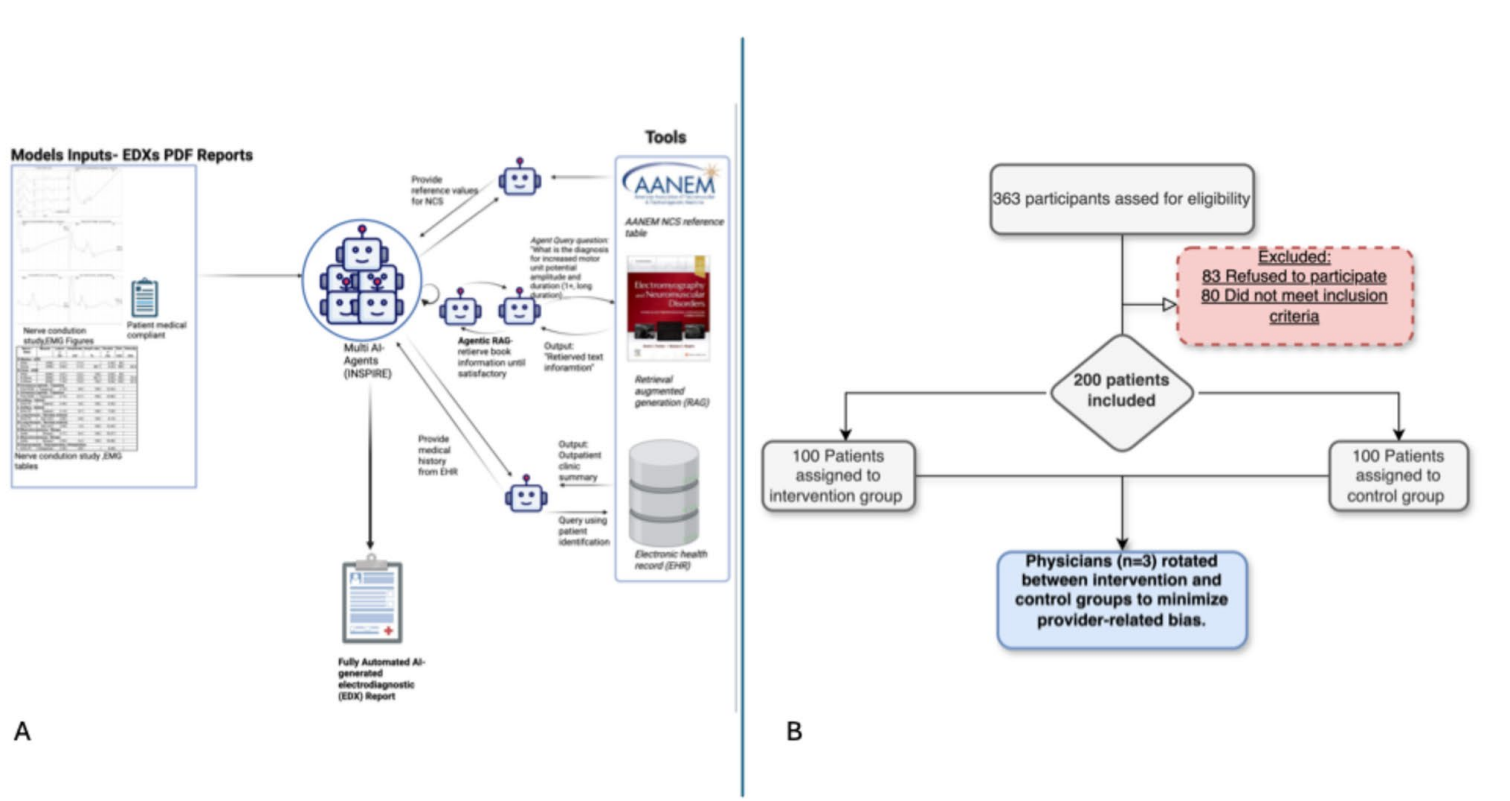
AI multi-agent architecture and CONSORT flow diagram of patient enrollment and allocation. **A** Multi-artificial intelligence (AI) agent system (INSPIRE). This approach begins with an electrodiagnostic (EDX) PDF report that includes the patient’s chief complaint, nerve conduction study (NCS) and EMG tables, and respective figures. The system then applies its “agentic” tools specifically, agentic retrieval-augmented generation (RAG) to query Electromyography and Neuromuscular Disorders: Clinical–Electrophysiologic–Ultrasound Correlations, 4th Edition until no further information is necessary; reference values extracted from AANEM guidelines; and the patient’s medical history retrieved from the electronic health record (EHR) to generate a preliminary EDX report aligned with recognized standards to the patient’s. **B** CONSORT flow diagram for patient enrollment, randomization, and allocation. Of 363 participants screened, 83 declined participation and 80 did not meet inclusion criteria; 200 participants were thus included and randomized to either the intervention group (AI + physician; *n* = 100) or the control group (physician only; *n* = 100). Three physicians were rotated between the two groups to reduce provider-related bias

The AI framework (INSPIRE) is built upon a multi agent framework rather than a single monolithic LLM (ChatGPT, for example). An agent is an autonomous instance of the language model governed by a role-specific prompt and a brief working memory; this modular design lets complex reporting be decomposed into focused sub-tasks. The agent’s framework allow iteratively exchange structured messages until they agree on a draft report. All agents run on the same ChatGPT-4o-class model securely deployed through Microsoft Azure OpenAI services. We used the library AutoGen 0.4 to develop a multi-AI agent system. The framework utilizes a bidirectional sequential multi-agent system using the SelectorGroupChat which implements a team, where participants take turns broadcasting messages to all other members. A generative model (LLM) selects the next speaker based on the shared context, enabling dynamic, context-aware collaboration. The model receives inputs of patient demographic data and EDX test (Tables and Figure). The model utilized through agentic retrieval augmented generation (RAG), by extracting information from the book Electromyography and Neuromuscular Disorders: Clinical–Electrophysiologic–Ultrasound Correlations 4th Edition. The agentic RAG operates by utilizing the agent query to initiate searches within the RAG framework. If the initial results are inadequate, the agent refine the querying until it acquires sufficient knowledge to adequately interpret the report (Fig. 2A).

### Outcomes (primary and secondary)

The primary analysis is the quality of the report based upon AIGERS score. Secondary outcomes were to compare the physician–AI integration through physician collaboration score, Physician compliance through the survey.

### Statistical analysis

Comparisons among AI, AI-physician (intervention), and physician-only (control) groups were performed using the Kruskal–Wallis test for non-parametric distributions, as verified by the Shapiro–Wilk normality test. Post-hoc pairwise comparisons between intervention and control groups were conducted using the Mann–Whitney *U* test. Inter-rater reliability among the three evaluating physicians (MK, GK, IT) was assessed using Fleiss kappa, while agreement between AI–physician and physician-only groups scores was measured using Cohen's kappa. For Clinical Diagnosis scores, we normalized values from the original − 5 to 0 range to a 0–1 scale. The AIGERS score was calculated as a weighted combination of the Finding Score (50%) and normalized Clinical Diagnosis Score (50%). For physician‐reported AI integration rating score (PAIR) scores, Spearman's rank correlation coefficients were calculated to evaluate relationships with AIGERS, PAIR, and physician compliance survey. The threshold for statistical significance was set at *p* < 0.05 for all analyses.

### Standard protocol approvals, registrations, and patient consents

The study adhered to CONSORT-AI Extension guidelines for reporting [22]. This study was approved by the Rambam Healthcare Campus institutional review board. All participants provided written informed consent. ClinicalTrials.gov Identifier: NCT06902675. MyTrial.gov registration: MOH_2025-01-22_013868.

## Results

### Baseline demographic and electrodiagnostic and clinical characteristics

Between January 21, 2025, and February 21, 2025, 100 participants (50%) were randomized to the intervention (AI-assisted EDX interpretation) group, and 100 (50%) were randomized to the control (standard EDX interpretation) group (Fig. 2B) by 1:1 ratio. In the intervention group, participants were included into both subgroups: the AI report-only group, which received reports generated solely by AI before any physician review (first stage), and the AI–physician group, where the AI-generated reports were reviewed by a physician (second stage). The demographic information for the three physicians included 33% male (*n* = 1), with a mean age of 51.66 years, all identifying as white Caucasian (*n* = 3), and an average of 16.6 years of experience in conducting electromyography (EDX). Baseline demographic and clinical characteristics were similar between groups (Table 1). The median age was 55.0 years (IQR 43.0–68.0) in the intervention group and 51.0 years (IQR 38.0–62.0) in the control group (*p* = 0.072). Both groups had similar proportions of male participants (56% intervention vs. 46% control; *p* = 0.15) and similar distributions of abnormal (75% vs. 71%; *p* = 0.52) and normal EDX findings (25% vs. 29%; *p* = 0.52). The majority of patients, 73% had abnormal EDX findings, with a similar distribution between the AI-assisted group (75%) and the physician-only group (71%), showing no significant difference between them. Among the specific diagnoses made, radiculopathy was the most common (34%), followed by mononeuropathy (32%) and polyneuropathy (13%). Less frequent diagnoses included plexopathy and myopathy, each seen in only 1% of patients, and there were no cases of motor neuron disease reported.

**Table 1:** Baseline demographic and clinical characteristics of patient electrodiagnostic reports

Features	Total (*n* = 200)	Intervention (AI–physician) (*n* = 100)	Control (Physician) (*n* = 100)	*p* value
Age [median, IQR]	53.0 [40.0–65.0]	55.0 [43.0–68.0]	51.0 [38.0–62.0]	0.072
Male (n, %)	102 (51.0%)	56 (56.0%)	46 (46.0%)	0.157
Abnormal tests	146 (73.0%)	75 (75.0%)	71 (71.0%)	0.522
Normal tests	54 (27.0%)	25 (25.0%)	29 (29.0%)	0.522
Correctness of AI model of detecting abnormality tests	–	70 (93.3%)	–	–
Correctness of AI model of detecting normal tests	–	23 (92.0%)	–	–
Diagnosis found in the reports				
Polyneuropathy diagnosis	26 (13.0%)	13 (13.0%)	13 (13.0%)	1.000
Mononeuropathy diagnosis	64 (32.0%)	32 (32.0%)	32 (32.0%)	1.000
Radiculopathy	68 (34.0%)	36 (36.0%)	32 (32.0%)	0.548
Plexopathy	2 (1.0%)	1 (1.0%)	1 (1.0%)	1.000
Myopathy	2 (1.0%)	1 (1.0%)	1 (1.0%)	1.000
Motor neuron disease	0 (0.0%)	0 (0.0%)	0 (0.0%)	–
Patient Comorbidities				
Diabetes mellitus	4 (2.0%)	2 (2.0%)	2 (2.0%)	1.000
Dyslipidemia	20 (10.0%)	10 (10.0%)	10 (10.0%)	1.000
Hypertension	18 (9.0%)	9 (9.0%)	9 (9.0%)	1.000
Congestive heart failure	0 (0.0%)	0 (0.0%)	0 (0.0%)	–
Ischemic heart disease	4 (2.0%)	2 (2.0%)	2 (2.0%)	1.000
Cancer	8 (4.0%)	4 (4.0%)	4 (4.0%)	1.000

In the intervention arm, the AI achieved 93.3% sensitivity for detecting abnormal studies and 92.0% specificity for confirming normal ones. These findings suggest that the diagnostic spectrum was well-balanced between groups and that the AI system was reasonably accurate in distinguishing normal from abnormal studies. Inaccuracies in identifying normal tests as abnormal arise from the AI model's tendency to classify very mild variations as abnormal or borderline.

### Primary outcome

**Fig. 3:**
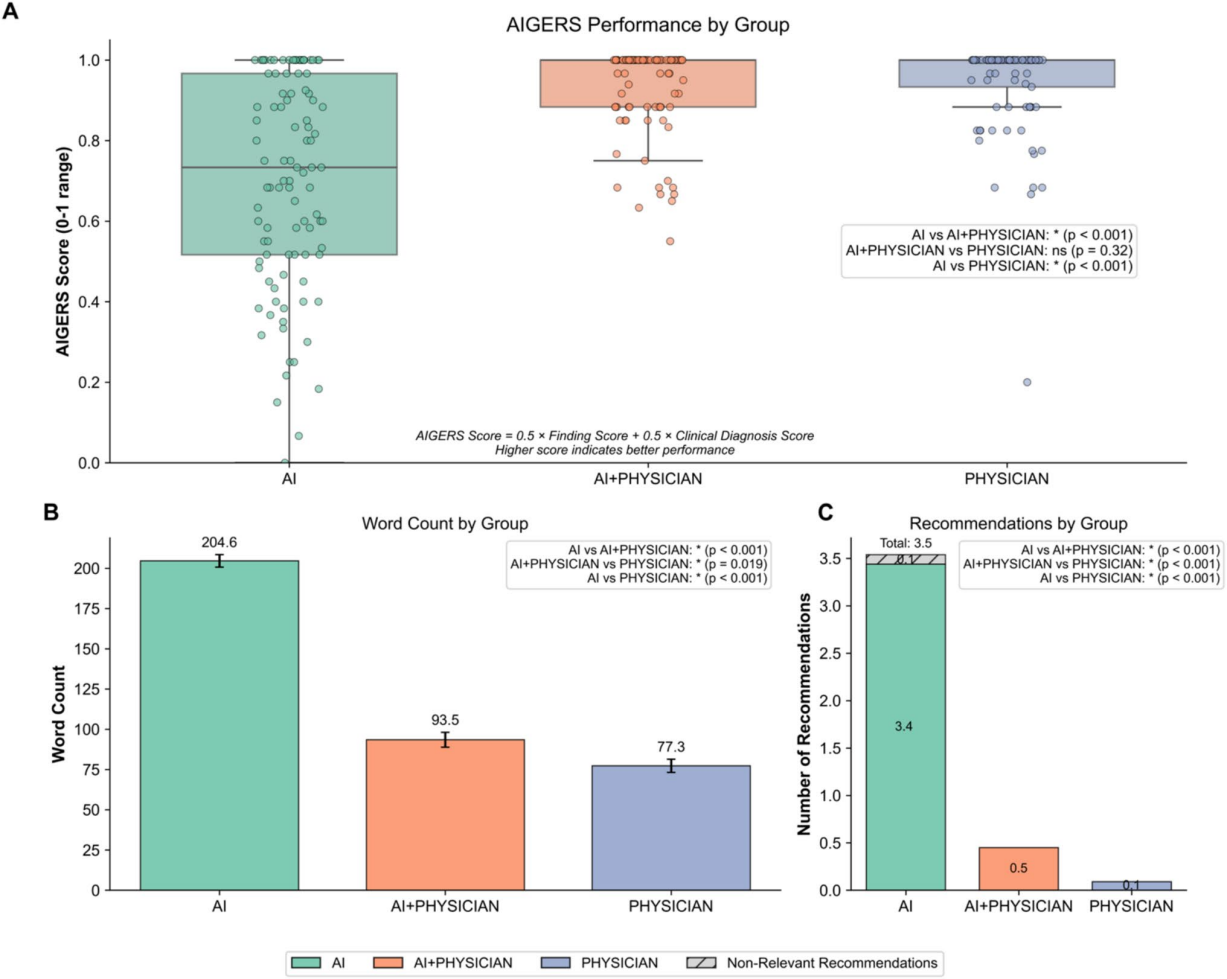
Comparative outcomes among AI, AI + physician, and physician-only arms description. **A** AIGERS (AI-Generated EDX Report Score) for each group on a 0–1 scale, where higher scores indicate better report quality. AIGERS is calculated as the mean of the finding score and clinical diagnostic accuracy score. **B** Mean word counts (± SE) by group, illustrating differences in report length among the AI-only, AI + physician, and physician-only arms. **C** Mean number of recommendations (both relevant and nonrelevant) included in the final reports by each group. Pairwise statistical comparisons are reported (e.g., *P* <.001) to indicate significant differences across groups

The primary outcome, assessed by the AI-Generated EMG Report Score (AIGERS) demonstrated no significant difference between the AI-assisted physician group (0.94 ± 0.11) and the physician-only group (0.94 ± 0.13, *p* = 0.32). Similarly, the two groups did not differ significantly in terms of individual AIGERS subcomponents, including the Finding Score (0.92 ± 0.14 vs. 0.92 ± 0.16; *p* = 0.43) and the Clinical Diagnosis Score (0.95 ± 0.09 vs. 0.95 ± 0.11; *p* = 0.51). AI model alone achieved significantly lower performance compared with both AI-assisted and physician-only reports across all metrics (AIGERS score: 0.70 ± 0.26; finding score: 0.70 ± 0.27; Clinical Diagnosis Score: 0.71 ± 0.29; three-way comparison *p* < 0.001 for all metrics). The control group showed substantial agreement assessed by the kappa score (ranging from 0.74 to 0.76). Kappa score decreased as AI usage was more prominent, intervention group with Fleiss kappa ranging from 0.67 to 0.69 and for AI reports solely 0.32–0.33, suggesting the nature of inconsistency regarding AI reports, while humans maintain more objective consistency (Table 2). When inspecting the distribution of performance scores, it further highlights similar performance between the AI-assisted physician and physician-only groups, with the AI-alone group demonstrating larger variability, reflecting inherent uncertainty in AI interpretation accuracy (Fig. 3).

**Table 2:** Models'performance differences

Features	AI	Fleiss kappa	AI–physician	Fleiss kappa	Cohen Kappa#	Physician	Fleiss kappa	Three-way *p* value	*p* value intervention vs. control
Finding Score (0–1 range)	0.70 ± 0.27	0.32	0.92 ± 0.14	0.69	0.12	0.92 ± 0.16	0.76	** < 0.001**	0.43
Clinical diagnosis score (− 5 to 0 range)	− 1.45 ± 1.45	0.33	− 0.24 ± 0.44	0.67	0.08	− 0.24 ± 0.53	0.74	** < 0.001**	0.51
Clinical diagnosis score (Normalized 0–1 range) †	0.71 ± 0.29	0.33	0.95 ± 0.09	0.67	0.08	0.95 ± 0.11	0.74	** < 0.001**	0.51
AIGERS SCORE ‡	0.70 ± 0.26	0.32	0.94 ± 0.11	0.68	0.1	0.94 ± 0.13	0.75	** < 0.001**	0.32
Report word count	204.60 ± 38.35	–	93.47 ± 46.05	–	–	77.29 ± 40.83	–	** < 0.001**	**0.019**
Report recommendations numbers	3.54 ± 0.92	–	0.5 ± 0.62	–	–	0.09 ± 0.29	–	** < 0.001**	** < 0.001**
Report non relevant recommendations numbers	0.10 ± 0.3	–	0	–	–	0	–	** < 0.001**	–

Values are presented as mean ± standard deviation. The Fleiss kappa values represent inter-rater reliability between three physicians (MK, GK, IT) who scored each report. Three-way *p* values were calculated using Kruskal–Wallis tests across all groups. The intervention vs. control column shows *p* values for direct comparison between AI–physician (intervention) and physician-only (control) groups

^*^Statistically significant (*p* < 0.05)

†Clinical Diagnosis Score normalized from original − 5 to 0 range to 0–1 scale using the formula: normalized score = (original score + 5)/5

‡AIGERS score calculated as 50% Finding Score + 50% normalized Clinical Diagnosis Score # Cohen's kappa values represent agreement between the AI and AI–physician groups

Bold are statistically significant

**Fig. 4:**
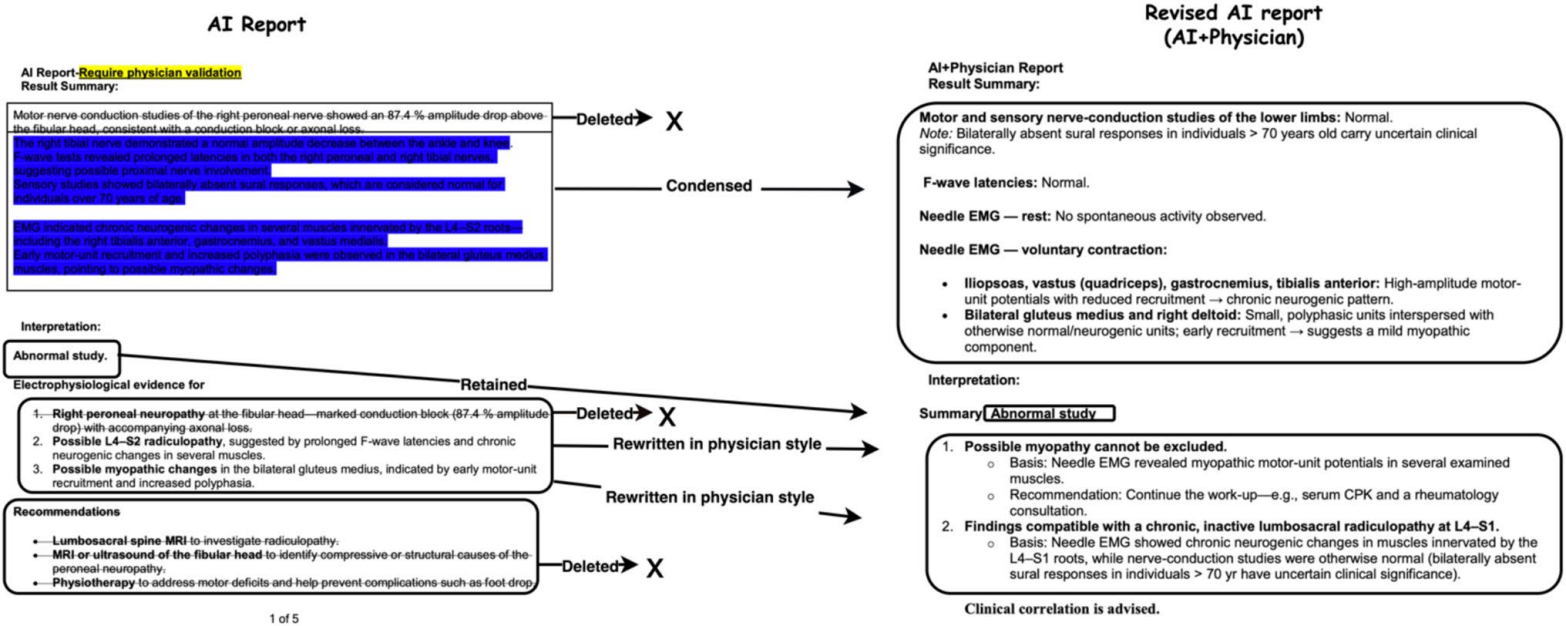
Example of an electrodiagnostic (EDX) report before and after physician revision. Left panel, *AI report*, shows the draft generated by the INSPIRE multi-agent large-language-model system; right panel, *revised AI report (AI* + *Physician)*, shows the final version after physician editing. Blue‐highlighted text in the left panel marks sentences that were modified during revision. Arrows indicate the physician’s action applied to each segment: deleted (text struck through and removed), condensed and simplified (shortened for clarity), rewritten in physician style (rephrased to match customary reporting language), and retained (left unchanged). This side-by-side excerpt illustrates typical edits made during the hybrid workflow; full-length reports are provided in the supplementary appendix

Notably, the AI‐generated reports were often more comprehensive than those produced by physicians (word count 204.60 ± 38.35 AI, 93.47 ± 46.05 AI–physician, 77.29 ± 40.83 physician control, < 0.001), as they often included detailed explanations distinguishing normal from abnormal findings and offered significantly more recommendations for future management (3.54 ± 0.92 AI, 0.5 ± 0.62 AI–physician, 0.09 ± 0.29 physician control, *p* < 0.001). In contrast, the standard physician reports tended to include only brief statements describing pathological findings and a suspected diagnosis, generally omitting suggestions or follow‐up steps for the primary care physician. Despite the AI’s more in‐depth content, physicians routinely removed most of its recommendations (3.54 ± 0.92 AI, 0.5 ± 0.62 AI–physician, *p* < 0.001) and explanatory text before finalizing the report, indicating limited compliance with the novel tool and highlighting potential barriers to its wider clinical adoption (Fig. 4).

### Secondary outcome

During the trial, we assessed how well physicians felt the AI system fit into their workflow using quick surveys after each EDX report and a final satisfaction questionnaire.

Physicians said at the end of the trial that the AI-tool improved the quality of their reports and made their work feel more professional, both scoring a 4.0 out of 5. They expressed above moderate trust in the AI's findings and diagnostic suggestions, with scores averaging 3.7 ± 0.6. While doctors found the collaboration with the AI-tool generally helpful (3.3 ± 0.6) and found it moderately reasonable to correct its mistakes (3.3 ± 0.6), these aspects showed room for improvement compared to the high scores on report quality and professionalism. However, the AI tool faced challenges in terms of efficiency (2 ± 1.7), ease of use (1.7 ± 1.5), and workload reduction (1.7 ± 1.2). Physicians attributed these low scores to an uncomfortable user interface and the lack of integration within their reporting tools, which led to various technical issues. Interestingly, satisfaction with the AI was not linked to how much revision was needed. These findings suggest that while AI has the potential for high compliance, technical challenges must be addressed before its integration into clinical practice (Supplementary Table 1).

## Discussion

Our model demonstrated high accuracy in identifying abnormal (93.3%) and normal (92.0%) test results within the intervention group. These findings suggest that the AI system was reasonably accurate in distinguishing normal from abnormal studies. Inaccuracies in identifying normal tests as abnormal arose from the AI model's tendency to classify very mild variations as abnormal or borderline. The RCT revealed that the multi-AI agent tool (INSPIRE) did not significantly enhance neurologists'performance in writing EDX reports compared to physicians working independently. Notably, the AI tool on its own performed substantially worse (*p* < 0.001), indicating that the combined physician–AI approach did not provide any additional benefit. A likely explanation beside subpar AI performance is low physician engagement, driven by the AI tool poor usability interface. Survey results showed low scores: ease of use (1.7), workflow efficiency (2.0), and workload reduction (1.7), suggesting the tool was not designed for physicians'needs. LLMs like this are often built for broad, general use. However, when integrating them into clinical practice, especially in neurology, it is critical to create a user-friendly interface tailored to physicians [23, 24]. Ideally, such tools should be embedded directly into the electronic health record (EHR) to enable seamless and efficient use. [25]

Physicians in this study had to manually revise LLM-generated text to meet medical documentation standards, consuming considerable amounts of time that could have been used for more reports. Similar challenges appeared in other studies [26] with AI-generated patient replies requiring significant revision due to a lack of empathy, poor personalization, and overly long messages. Our trial found the same issues, including lengthy drafts (*p* < 0.001) and generic advice (*p* < 0.001), like recommending weight-loss programs for all patients with a BMI over 30.

The place where such AI-tool could shine in the EDX field is by reducing the burden and workload of routine normal and straightforward cases such as mononeuropathy diagnoses. Notably, we found that AI‐generated documentation was often more thorough and well‐structured, potentially improving clarity for both clinicians and patients. Our framework only misclassified two cases that were indeed normal as abnormal, and similarly misidentified five abnormal cases as normal. These discrepancies primarily occurred with borderline test results, highlighting the necessity for final interpretation by a physician. The AI tool could be seamlessly integrated into clinical practice, activated primarily for simple cases, with an output that generates a final report for review within the EHR system. This would allow physicians to provide the ultimate verification of the results [27]. There was agreement among the physicians who participated in the trial regarding such a notion. By alleviating the burden of crafting EDX reports from scratch and interpreting routine tests, tasks that AI can perform with high sensitivity, workflow efficiency can significantly be enhanced. These findings are particularly relevant now that many health systems offer Health Insurance Portability and Accountability Act—compliant chatbots that physicians can use in clinical settings, often with no to minimal training on how to use these tools. [28]

One of the main goals of our RCT was to investigate the dynamics between AI-driven systems and physicians, assessing whether clinical practice should favor a singular approach or facilitate a collaborative interface between both parties. A recent meta-analysis has shown that human–AI systems often underperform compared to either humans or AI operating independently [29]. The study suggested that while AI shows considerable potential for tasks involving creativity and documentation, it struggles with decision-making responsibilities. This implies a more effective use of AI in support roles, such as assisting physicians with routine documentation, rather than relying on it for diagnostic responsibilities. This aligns with the results of this study, where the diagnostic performance was subpar to the report generative capabilities. This aligns with other medical studies that have explored the integration of AI with physicians, demonstrating promising outcomes [13, 14, 16]. By acting as a secondary reviewer and pre-screening images, AI has effectively reduced the workload for medical image interpretation [16]. This is similar to our proposal for streamlining basic EDX reports.

It is important to take notice that EDX tests are in their nature complicated task to infer. The field of medical imaging reached high accuracy due to the rise of vision language model [30]. In contrast to the advancements seen in medical imaging, where vision-language models have achieved high accuracy, the EDX domain necessitates the integration and interpretation of a diverse array of multimodal inputs [31]. The limited body of research beyond medical imaging and solely text format creates challenges in evaluating the efficacy of LLMs in clinical diagnostics that necessitate a multi-modal approach. Building on our earlier work, which showed that partitioning the task among multiple agents outperformed a single end-to-end language model [32], we adopted a multi-agent architecture. Even so, the present trial demonstrates that a conventional, LLM whether single or multi-agent still falls short of expert performance. True parity may require next-generation “reasoning” models (e.g., ChatGPT o3) LLMs fine-tuned with an additional objective that forces them to generate a chain-of-thought and verify intermediate steps before issuing a final answer [33, 34]. Therefore, this study showcases that traditional LLM without reasoning in a multi agent format is not compatible with replacing physicians in interpreting EDX tests.

This study has a few limitations. First, only 3 physicians participated, which may have introduced bias regarding the use of the AI tool in everyday practice. Second, the AI framework did not incorporate a robust reasoning model or advanced machine‐learning and deep‐learning techniques to handle numerical data a known limitation of current LLMs [35, 36]. Third, our sample size was insufficient to detect a significant difference.

In conclusion, our findings showed diagnostic performance was not changed using integrated AI–human in its current form. A consistent barrier is low trust in AI outputs. Surveys show many physicians are disinclined to use recommendations from a “black-box” model that they do not fully trust or cannot understand. [37, 38] Showing the reasoning for each of the reporting elements would have slowed the process significantly and even enhance cognitive fatigue [39]. In addition, we believe that the integration of any digital tool into clinical workflow is an obstacle to compliance. If using the AI requires extra steps or disrupts routine practice, busy clinicians may simply bypass it. Effective decision support must blend seamlessly into existing workflows with intuitive interfaces. [40] Finally, physicians are pragmatic and more likely to adopt an AI recommendation if it demonstrably adds value to patient care. Conversely, if the clinical benefit of the AI is unclear or marginal, compliance drops. Prior trials have shown that clinicians favor AI tools that clearly outperform usual clinical judgment, while they will dismiss even a highly sophisticated model that offers no improvement over standard practice [40]. Future studies should examine larger randomized controlled trials in fields beyond medical imaging, exploring how LLMs might be effectively integrated into clinical workflows to complement, rather than supplant, physician expertise.

## Supplementary Information

Below is the link to the electronic supplementary material.

Supplementary file1 (PDF 1871 KB)

Supplementary file2 (DOCX 33 KB)

## Data Availability

The data underlying this article will be shared on reasonable request to the corresponding author. Anonymized data not published within this article are available by request.

## Code availability

Data processing, development, and INSPIRE analyses were conducted using built-in functions in Python programming language. The custom code can be provided upon reasonable request to the corresponding author.
