# Supplementary material for: AI-Based EMG Reporting: A Randomized Controlled Trial
Source: J Neurol. 2025 Aug 22;272(9):586. doi: 10.1007/s00415-025-13261-3 (PMC12373542; doi:10.1007/s00415-025-13261-3)
Supplement: Supplementary file 1 — Supplementary file1 (PDF 1871 KB) [file 415_2025_13261_MOESM1_ESM.pdf]

Supplementary Table 1. Physician Assessment of AI Integration in EDX Reporting

Assessment Domain	Score (Mean ± SD)
Physician Perception (1-5 scale)	
**Report quality enhancement**	4.0 ± 0.0
**Trust in AI findings**	3.7 ± 0.6
**Trust in AI diagnoses**	3.7 ± 0.6
**Contribution to professionalism**	4.0 ± 0.0
**Effectiveness of collaboration**	3.3 ± 0.6
**Ability to correct errors**	3.3 ± 0.6
**Ease of use**	1.7 ± 1.5
**Workflow efficiency**	2.0 ± 1.7
**Workload reduction**	1.7 ± 1.2
**Overall satisfaction**	2.3 ± 1.5
Scores (0-1 scale)	
**Report Usability (RU)**	0.82 ± 0.20
**Error Fixes (EF)**	0.82 ± 0.22
**Trust in Report (TCR)**	0.81 ± 0.30
**Revision Effort (RE)**	0.65 ± 0.30
**Overall PAIR Score**	0.78 ± 0.23
Correlation: Score vs Quality	**Spearman's ρ (p-value)**
**PAIR Score vs AIGERS**	0.03 (0.77)
**PAIR Score vs Finding Score**	0.03 (0.77)
**PAIR Score vs Clinical Diagnosis**	0.04 (0.69)
**Trust in Report vs AIGERS**	0.11 (0.28)

Note: Physician Perception data collected from 3 physicians who participated in the clinical trial, rating their experience with the AI system. Physician-AI Interaction scores derived from 100 individual report assessments. Correlation analysis performed using Spearman's rank correlation due to non-normal distribution of data. Higher values indicate better performance except for revision Effort, where higher scores indicate greater effort required.

Supplementary Figures

Figure 1. Comparison of AI, Physician, and Combined AI–Physician EDX Performance.

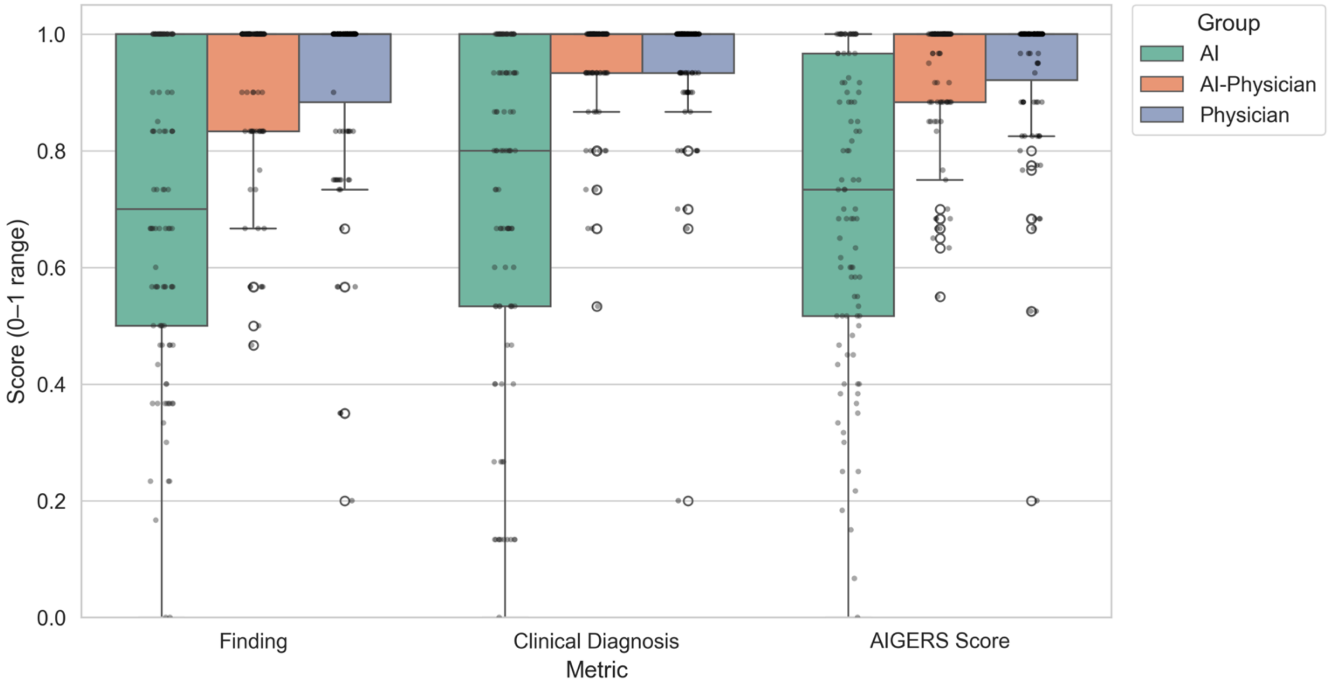

Box-and-whisker plots depict performance distributions for each interpretation modality on three metrics: Findings, Clinical Diagnosis, and AIGERS (range, 0 to 1). AI alone (green) demonstrates variable performance, with notable improvements when integrated with a physician (orange). Physician-only results (blue) are shown for comparison. Horizontal lines within each box indicate the median, with whiskers and dots representing variability and outliers.

Figure 2. Mean Physician Ratings by Domain and Percentage of Positive Ratings

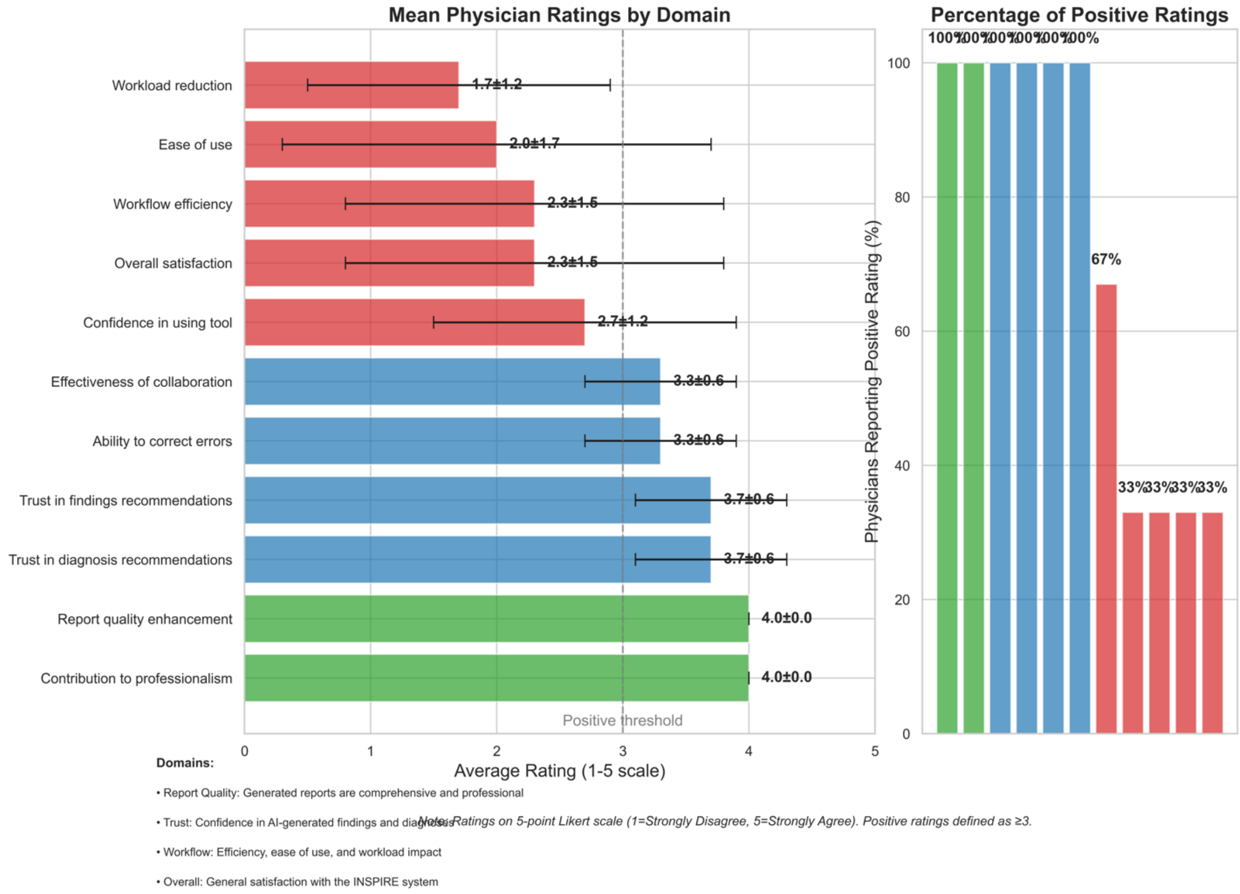

The bar chart (left) summarizes physician responses (±95% CI) on a 5-point Likert scale (1=Strongly Disagree, 5=Strongly Agree) across 10 usability and satisfaction domains: workload reduction, ease of use, workflow efficiency, overall satisfaction, confidence in using the tool, effectiveness of collaboration, ability to correct errors, trust in findings recommendations, trust in diagnosis recommendations, report quality enhancement, and contribution to professionalism. The vertical dashed line marks the positive threshold (≥*3*). The bar chart (right) shows the percentage of physicians reporting positive ratings in each domain, ranging from 33% (for ease of use and workload reduction) to 100% (for trust in findings and diagnosis recommendations, report quality, and professionalism)

Figure 3. Relationship Between Physician Characteristics and Acceptance of AI-Assisted tool

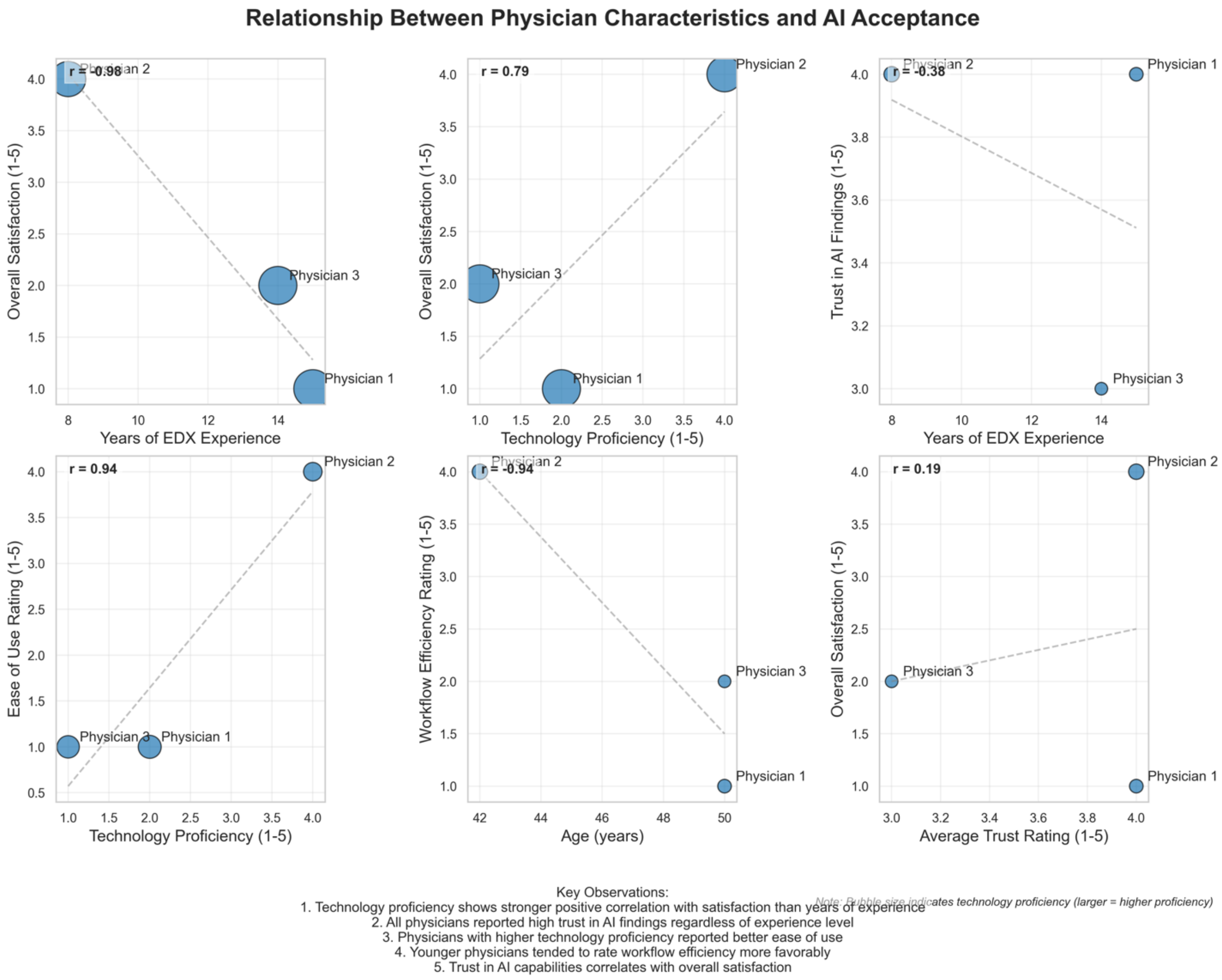

Six scatterplots depict correlations between physicians' years of electromyography (EDX) experience, age, technology proficiency (scale 1–5), and key outcomes (overall satisfaction, trust in AI findings, ease of use, workflow efficiency). Larger circles represent higher satisfaction or trust ratings, with each circle labeled by physician identifier. Dashed trend lines indicate correlation strength (Pearson *r* values). Key observations include higher technology proficiency correlating more strongly with satisfaction compared with years of experience, consistently high trust in AI across all experience levels, and a tendency for younger physicians to rate workflow efficiency more favorably.

Figure 4. Word Count vs. Recommendations in AI, AI+Physician, and Physician-Only Reports

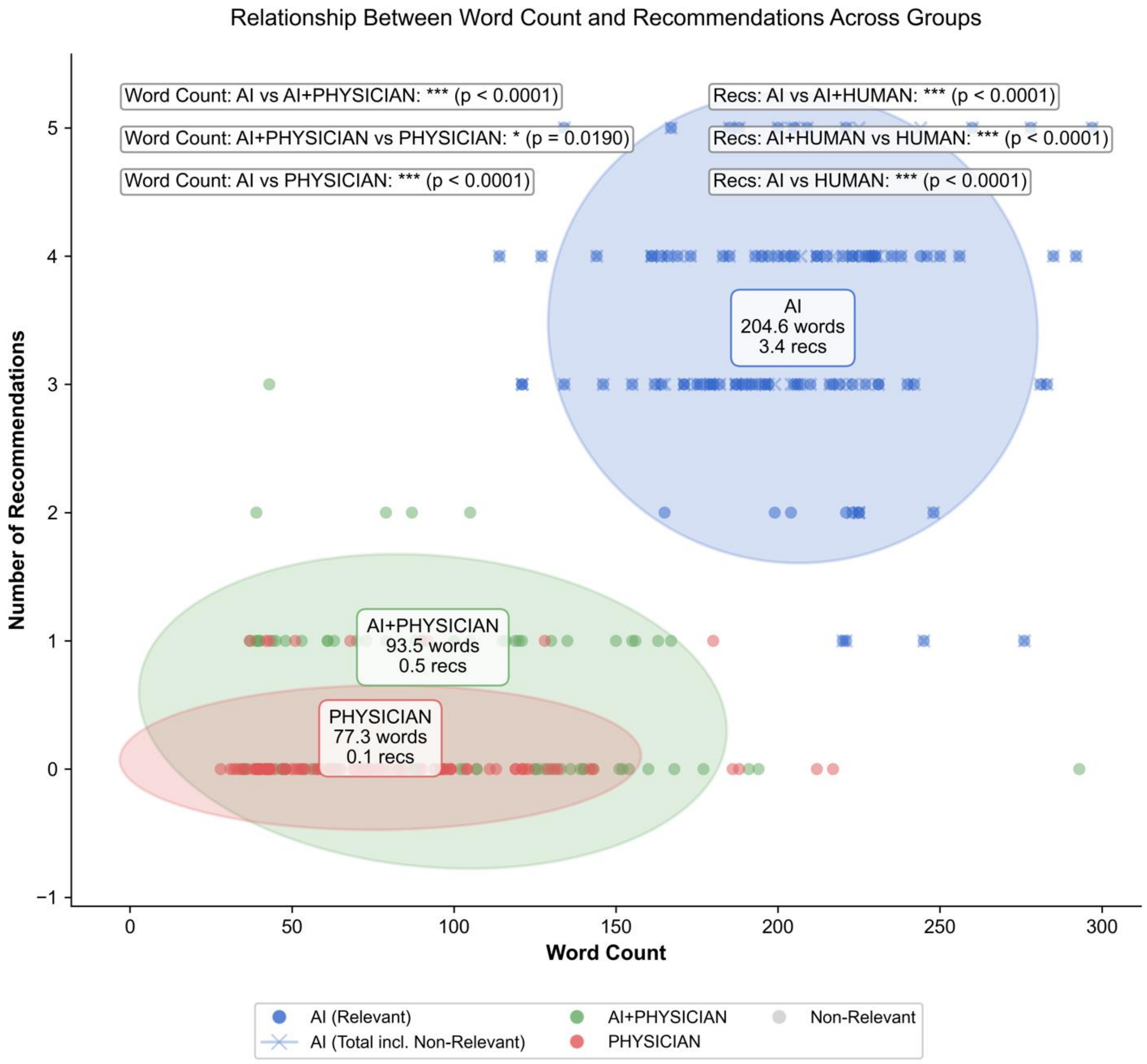

Scatterplot shows each report's total word count (x-axis) versus number of recommended next steps (y-axis), with ellipses indicating the distribution of each study arm: AI-only (blue), AI+physician (green), and physician-only (red). AI-generated reports (mean 204.6 words, 3.4 recommendations) were significantly longer and contained more recommendations than AI+physician (93.5 words, 0.5 recommendations) or physician-only (77.3 words, 0.1 recommendations) reports (*P*<.001). Physicians commonly removed non-relevant or excessive AI suggestions before finalizing the report (non-relevant recommendations shown in gray).

Finding grading sheet

F-Score	Description	Example
**0**	Missed most findings in the original report.	Fails to mention critical findings like radiculopathy or neuropathy entirely.
**0.5**	Missed major findings but captured some.	Captures neuropathy but misses radiculopathy findings.
**1**	Captured all findings from the original report.	Accurately identifies all findings present in the original report.
**-0.3**	Capture mistaken findings alongside	Identify prolong peroneal latency depsite being normal

CD score grading sheet

CD-Score	Scenario	Description	Example
-5	Defines normal when pathological or vice versa	Fails to recognize significant pathology and incorrectly labels the study as normal.	Missing radiculopathy completely and reporting as normal.
-3	Diagnoses incorrectly	Provides a wrong diagnosis that could mislead clinical care.	Diagnosing neuropathy when the true finding is radiculopathy.
-1	partial diagnosis/included additional non-related diagnosis/ non-specific diagnosis	Fails to identify one or more key findings from the original report.	Add mild right ulnar neuropathy to C8-T1 radiclopathy/ diagnose C8-T1 radiculopathy instead of C8 radiculopathy
0	Diagnoses exactly	Matches the original report, correctly identifying all findings.	Diagnoses chronic C7 radiculopathy as stated in the original report.

Evaluation of the Findings and Clinical Diagnosis scores involved a manual review of the three types of report (AI-Physician, AI, Human). This review process was conducted by three senior neuromuscular specialists (M.K., G.K.M I.T), all three physicians were blinded to the group of the report and did not practice as part of the clinical trial itself, ensuring consistency and lack of bias. The study was considered normal if all AANEM NCS data were within normal limits apart from distal sensory responses in the sural nerve among individuals aged over 70 years.

PAIR score

To evaluate the quality of physician–AI collaboration in AI-generated EDX reports we assessed through a survey containing four questions(Q1-Q4) (25% weight) each scored from 0 to 1. Q1, focused on Revisions, evaluated the amount of revision effort required from the physicians. Q2 dealt with Text Fixes, measuring how much editing was necessary to improve the report's wording. Q3, concerning Report Usability, looked at how practical and comprehensive the report was from the physicians' perspective. Lastly, Q4 examined Trust in the AI report, gauging the confidence physicians had in the generated results. A higher score (closer to 1) indicates fewer modifications needed (Q1, Q2, Q3), fewer hallucinations (Q3, Q4), and a stronger alignment with physician standards (Q2, Q3). The detailed scoring rubric can be found in the Supplementary materials.

In addition, physicians took a survey at the end of the trial. These scores were correlated to the mean AIGERS and collaboration score of the reports the physician was tasked to cooperate with.

Statistical analysis

Post-hoc pairwise comparisons between intervention and control groups were conducted using the Mann-Whitney U test. Inter-rater reliability among the three evaluating physicians (MK, GK, IT) was assessed using Fleiss kappa, while agreement between AI-Physician and Physician-only groups scores was measured using Cohen's kappa. For Clinical Diagnosis scores, we normalized values from the original -5 to 0 range to a 0-1 scale. The AIGERS score was calculated as a weighted combination of the Finding Score (50%) and normalized Clinical Diagnosis Score (50%). For physician-reported AI integration rating score (PAIR) scores, Spearman's rank correlation coefficients were calculated to evaluate relationships with AIGERS, PAIR, and physician compliance survey. The threshold for statistical significance was set at $p<0.05$ for all analyses.

Instructions for Completing the PAIR Score Evaluation

Purpose:

This evaluation is designed to assess the integration of AI-generated reports into your clinical workflow. The PAIR Score evaluates the effort, accuracy, usability, and trustworthiness of the AI-generated report. Your feedback will help us improve the system's performance and better integrate AI into clinical practice.

Physician Evaluation Form

Patient ID: ______________________

Patient Report first four id letters______________________

Date of Review: ______________________

---

1. Revision Effort (RE)

Question: How much effort and time were required to revise the AI-generated report?

Effort Level	Description	Score
Minimal Effort	Quick review with no significant changes.	1.0
Low Effort	Some review and minor adjustments.	0.8
Moderate Effort	Multiple corrections or longer review time.	0.5
High Effort	Extensive corrections requiring significant time.	0.0

Your Score: _____________

Comments (optional): ____

---

2. Error Fixes (EF)

Question: How many and what type of errors were identified and corrected in the report?

Error Level	Description	Score
No Errors	No corrections needed.	1.0
Minor Errors	Small corrections (e.g., formatting or minor details).	0.8

Moderate Errors	Multiple data inaccuracies or missing findings.	0.5
Critical Errors	Major errors, hallucinations, or misleading conclusions.	0.0

Your Score: ____________

Describe Any Errors: ____

---

3. Report Usability (RU)

Question: How practical and complete was the AI-generated report for clinical use?

Usability Level	Description	Score
Fully Usable	Clear, complete, and actionable.	1.0
Mostly Usable	Minor gaps or ambiguities.	0.8
Partially Usable	Moderate gaps or unclear conclusions.	0.5
Not Usable	Significant effort needed to interpret or rewrite.	0.0

Your Score: ____________

Comments (optional): ____

---

4. Trust in the Current Report (TCR)

Question: How confident are you in the findings, conclusions, and recommendations of this specific AI-generated report?

Confidence Level	Description	Score
Fully Confident	Trusts all aspects of the report.	1.0
Mostly Confident	Minor doubts about specific sections.	0.8
Neutral	Uncertain about some conclusions.	0.5
Not Confident	Requires extensive validation or corrections.	0.0

Your Score: ____________

Comments (optional): ____

Additional Comments:

Please provide any feedback on your experience with the AI system or suggestions for improvement.

Physician Compliance Survey-INSPIRE TRIAL

This survey is conducted to assess your experience and compliance with the AI tool for generating EDX reports. Please rate the following statements on a scale of 1 to 5, where:

1 = Strongly Disagree
2 = Disagree
3 = Neutral
4 = Agree
5 = Strongly Agree

Please complete the form at start of trial (Prior using INSPIRE), Middle of the trial (After you used INSPIRE), End of the Trial (After having enough confidence of using INPSIRE by yourself)

* Indicates required question

Baseline Questions

Please answer all baseline questions- answer once no need three times

1. State your name *

____

2. What is your gender

Mark only one oval.

- ◯ Male
- ◯ Female
- ◯ Other: _______________

3. Age

____

4. What is your current position?

Mark only one oval.

- ◯ Resident Physician
- ◯ Specialist Physician
- ◯ Senior Physician
- ◯ Other: ____________________

5. How many years of experience do you have in conducting EDX study

____

Baseline Knowledge and Experience

Questions regarding the knowledge of technology and AI-Tools

6. How would you rate your knowledge of technology on a scale of 1 to 5?

Mark only one oval.

1	2	3	4	5
◯	◯	◯	◯	◯

7. How familiar are you with AI-assisted tools prior to this trial?

Mark only one oval.

1	2	3	4	5
◯	◯	◯	◯	◯

8. Have you used any AI tools in clinical practice before this trial?

Mark only one oval.

- Yes
- No

Physician Compliance (INSPIRE) Questions-Answer Start, Middle, End

9. I find the AI tool easy to use.

Mark only one oval.

1	2	3	4	5

10. The AI tool improves the efficiency of my workflow.

Mark only one oval.

1	2	3	4	5

11. The AI tool enhances the quality of EDX reports.

Mark only one oval.

1	2	3	4	5

12. The AI tool reduces my overall workload.

Mark only one oval.

1	2	3	4	5
◯	◯	◯	◯	◯

13. I feel confident in using the AI tool.

Mark only one oval.

1	2	3	4	5
◯	◯	◯	◯	◯

14. Collaboration with the AI tool is effective.

Mark only one oval.

1	2	3	4	5
◯	◯	◯	◯	◯

15. I trust the AI tool's recommendations for clinical **findings (SUMMARY OF STUDY**) .

Mark only one oval.

1	2	3	4	5
◯	◯	◯	◯	◯

16. I trust the AI tool's recommendations for clinical **diagnosis (CLINICAL INTERPERTATION**) .

Mark only one oval.

	1	2	3	4	5
	◯	◯	◯	◯	◯

17. I can effectively correct errors in the AI-generated reports

Mark only one oval.

	1	2	3	4	5
	◯	◯	◯	◯	◯

18. The AI tool contributes to clearer and more professional EDX reports.

Mark only one oval.

	1	2	3	4	5
	◯	◯	◯	◯	◯

19. I am satisfied with the overall performance of the AI tool.

Mark only one oval.

	1	2	3	4	5
	◯	◯	◯	◯	◯

This content is neither created nor endorsed by Google.

Google Forms

Multi-Agent Architecture and Workflow

Agent Architecture

The system consists of 10 specialized agents working in a coordinated sequence:

1. **Patient Specialist Agent**: Analyzes patient history and reasons for referral to identify key clinical symptoms and determine the purpose of the EDX study. This agent establishes the clinical context for subsequent interpretation.
2. **NCS Analyst Agent**: Analyzes motor and sensory nerve conduction studies according to AANEM standards. This agent processes numerical values from the EDX tables, applies reference ranges, and classifies findings as normal or abnormal for each anatomical location.
3. **Figure Analyst Agent**: Interprets electrophysiological waveforms and patterns in EDX figures independently from tabular data. This agent focuses on visual representations of electrical activity to identify abnormalities not evident in numerical data alone.
4. **Integration Agent**: Correlates table findings, figure findings, and patient history to identify distribution patterns, evaluate chronicity, assess severity, and create a unified clinical picture. This agent performs the critical function of synthesizing multimodal information.
5. **Validation Agent**: Reviews the integrated findings for technical standard compliance, measurement accuracy, and interpretation consistency. This agent acts as an initial quality control checkpoint before proceeding to diagnostic interpretation.
6. **Query Specialist Agent**: Generates specific diagnostic queries based on the validated EDX findings to retrieve relevant medical knowledge. This agent structures questions to maximize the value of retrieved information.
7. **Query Critic Agent**: Evaluates the relevance and sufficiency of information retrieved from the medical knowledge tool. This agent ensures diagnostic reasoning is based on appropriate evidence and refines queries when necessary.
8. **Diagnostic Agent**: Formulates a comprehensive clinical interpretation using the validated findings and medical knowledge retrieved through queries. This agent applies neuromuscular expertise to translate electrophysiological data into clinically meaningful diagnoses.
9. **Critic Agent**: Validates the clinical diagnosis and reasoning through critical evaluation. This agent serves as a final quality control checkpoint before report generation.
10. **Report Generator Agent**: Creates a structured medical report including key findings, metrics, clinical interpretation, and recommendations. This agent formats information according to standardized reporting templates.

Workflow Pipeline

The INSPIRE framework operates through a bidirectional sequential workflow with distinct analysis and reporting phases:

1. **Analysis Phase**:
   - Patient history and clinical context evaluation (Patient Specialist)

- Table data processing (NCS Analyst)
- Figure interpretation (Figure Analyst)
- Integration of multimodal findings (Integration Agent)
- Validation of integrated interpretation (Validation Agent)
- Knowledge acquisition through query generation and evaluation (Query Specialist and Query Critic)
- Diagnostic reasoning and formulation (Diagnostic Agent)
- Critical evaluation of diagnostic conclusions (Critic Agent)

2. **Reporting Phase**:
   - Structured report generation (Report Generator)

The framework incorporates multiple feedback loops where critical agents can redirect the workflow back to previous stages for refinement. For example, the Validation Agent can return the process to the NCS Analyst if inconsistencies are detected, and the Query Critic can prompt the Query Specialist to refine inadequate queries.

Technical Implementation

The system is implemented using Microsoft Azure's AI platform with the AutoGen 0.4 library for multi-agent orchestration. The SelectorGroupChat module dynamically determines which agent should respond next based on the conversation context and current phase. The framework incorporates retrieval-augmented generation (RAG) capabilities through integration with medical reference materials, enhancing diagnostic accuracy through access to standardized electrophysiological knowledge.

All agents operate on the same underlying language model (GPT-4o) but with specialized system prompts that define their distinct roles, expertise, and decision-making parameters. This approach enables a single foundational model to perform diverse specialized functions within the coordinated diagnostic pipeline.
