# Supplementary material for: AI-Based EMG Reporting: A Randomized Controlled Trial
Source: J Neurol. 2025 Aug 22;272(9):586. doi: 10.1007/s00415-025-13261-3 (PMC12373542; doi:10.1007/s00415-025-13261-3)
Supplement: Supplementary file 2 — Supplementary file2 (DOCX 33 KB) [file 415_2025_13261_MOESM2_ESM.docx]

**Assessment of AI‑Assisted Neuromuscular EDX Reporting on Diagnostic Quality: The INSPIRE Trial Protocol**

**Version**: 1.0, January 2, 2025

**Abstract**

**Introduction**: Electrodiagnostic (EDX) testing is fundamental for diagnosing neuromuscular disorders. However, interpretation is time-consuming and requires specialized expertise. This trial aims to evaluate whether an AI-assisted approach can improve the accuracy and quality of EDX reports compared with conventional physician-only interpretation.

**Methods and Analysis**: This single-center randomized controlled trial will enroll 200 adult patients scheduled for routine EDX testing at Rambam Health Care Campus, Haifa, Israel between January 21, 2025, and February 21, 2025. Participants will be randomized 1:1 to either AI-assisted physician reporting or standard physician-only reporting. The AI intervention (INSPIRE) is a multi-agent framework built upon ChatGPT-4o large language models hosted on the AZURE AI platform, which will generate preliminary EDX reports for physician review and editing. The primary outcome is report quality, assessed using the AI-Generated EMG Report Score (AIGERS; range 0-1). Secondary outcomes include measures of AI-physician collaboration efficiency and physician satisfaction with the tool. A two-week calibration phase will precede trial initiation to optimize AI integration.

**Ethics and Dissemination**: This study was approved by the Rambam Healthcare Campus Institutional Review Board. Results will be published in peer-reviewed journals and presented at scientific conferences.

**Trial Registration**: ClinicalTrials.gov Identifier: [NCT06902675](https://clinicaltrials.gov/study/NCT06902675)

**Key Points**

- This trial evaluates the impact of AI-assisted interpretation on the quality of electrodiagnostic (EDX) reports compared to standard physician-only interpretation.
- The AI system (INSPIRE) is a multi-agent framework using LLMs to generate preliminary EDX reports that are then reviewed and edited by physicians.
- Outcomes include report quality (measured by the AIGERS score), efficiency metrics, and physician satisfaction with the AI tool.

**1. Administrative Information**

**1.1 Title**

Assessment of AI‑Assisted Neuromuscular EDX Reporting on Diagnostic Quality: A Randomized Controlled Trial Using a Multi-Agent LLM Framework (INSPIRE)

**1.2 Trial Registration**

ClinicalTrials.gov Identifier: [NCT06902675](https://clinicaltrials.gov/study/NCT06902675)
MyTrial.gov registration: [MOH_2025-01-22_013868](https://my.health.gov.il/CliniTrials/Pages/MOH_2025-01-22_013868.aspx).

**1.3 Protocol Version**

Version 1.0, January 2, 2025

**1.4 Funding**

No external funding was received for this study.

**1.5 Roles and Responsibilities**

**1.5.1 Protocol Contributors**

- Alon Gorenshtein: Study concept and design, protocol development, trial management
- Yana Weisblat MD: Clinical implementation, protocol development
- Mohamed Khateb MD PhD: Analysis plan development, protocol review
- Shahar Shelly MD: Study concept and design, protocol development, trial management, study supervision

**1.5.2 Trial Sponsor**

Not funded

**2. Introduction**

**2.1 Background and Rationale**

**2.1.1 Intended Use of the AI Intervention**

The INSPIRE AI framework is intended to assist neurologists in interpreting electrodiagnostic (EDX) tests (including electromyography and nerve conduction studies) and generating diagnostic reports. The primary purpose is to reduce documentation burden, standardize reporting, and potentially improve diagnostic accuracy. The intended users are board-certified neurologists who regularly perform EDX tests in clinical practice.

Within the clinical pathway, EDX studies are conducted to evaluate patients with suspected neuromuscular disorders. Currently, physicians manually interpret the raw data and compose reports, a process that is time-consuming and subject to variation in quality and completeness. The INSPIRE system will generate a preliminary report based on the raw EDX data, which will then be reviewed, verified, and edited by a neurologist before finalization. This is expected to streamline workflow while maintaining or improving diagnostic accuracy.

**2.1.2 Pre-existing Evidence for the AI Intervention**

The INSPIRE framework builds upon recent advances in large language models (LLMs) that have shown promise in clinical documentation and decision support. Prior to this trial, we conducted a retrospective study using old version of INSPIRE using retrospective EDX reports. This phase demonstrated the technical feasibility of the system to interpret EDX data and generate structured reports.

There is currently limited published evidence specifically evaluating AI for EDX interpretation. However, studies in other medical specialties have shown that AI can assist with clinical documentation and reduce physician workload. Our approach is novel in applying a multi-agent LLM architecture to this specific clinical task.

**2.2 Explanation for Choice of Comparators**

The control arm (physician-only reporting) represents the current standard of care for EDX interpretation. This comparator was chosen to directly assess whether the addition of AI assistance provides measurable improvements over existing clinical practice. By comparing AI-assisted reporting to standard physician reporting, we can determine the added value of the AI intervention in real-world clinical settings.

**2.3 Objectives**

Primary objective: To determine whether AI-assisted EDX interpretation improves the quality of diagnostic reports compared to standard physician-only interpretation.

Secondary objectives:

1. To assess the technical performance of the AI system (sensitivity, specificity) for detecting abnormal EDX findings
2. To evaluate physician satisfaction with the AI system
3. To assess the impact of AI assistance on documentation time and workflow
4. To identify specific areas where AI assistance is most beneficial in EDX reporting

**2.4 Trial Design**

This is a single-center, randomized controlled trial with two parallel arms and 1:1 allocation ratio:

1. Intervention arm: AI-assisted physician EDX report generation
2. Control arm: Standard physician-only EDX report generation

**3. Methods: Participants, Interventions, and Outcomes**

**3.1 Study Setting**

The trial will be conducted at the neuromuscular outpatient clinic of Rambam Health Care Campus, a tertiary care academic medical center. All participants will undergo EDX testing according to standard clinical protocols.

**3.1.1 Integration Requirements**

The implementation of the INSPIRE system requires the following:

Onsite requirements:

- Secure, HIPAA-compliant workstations with access to the AZURE AI platform
- Integration with the electronic health record (EHR) system for accessing patient history
- Dedicated secure server for data processing

Offsite requirements:

- Secure Azure cloud infrastructure for hosting the AI models
- Encrypted data transmission channels compliant with hospital IT security protocols
- Regular system maintenance and updates by the technical team

**3.2 Eligibility Criteria**

**3.2.1 Participant-Level Inclusion Criteria**

1. Age ≥18 years
2. Referred for routine EDX testing at the neuromuscular clinic
3. Able to provide informed consent
4. Scheduled for complete EDX evaluation including both nerve conduction studies and needle EMG

**3.2.2 Participant-Level Exclusion Criteria**

1. Age <18 years
2. Patients undergoing single-fiber EMG testing
3. Incomplete EDX testing (lacking either nerve conduction studies or needle EMG)
4. Inpatient status
5. Previous EDX testing within the past three months

**3.2.3 Input Data-Level Inclusion Criteria**

1. Complete nerve conduction study data including sensory and motor studies
2. Complete needle EMG examination of at least three muscles
3. High-quality recordings with minimal artifacts
4. All standard measurements recorded (amplitude, latency, conduction velocity)
5. Patient demographic data and clinical history available

**3.2.4 Input Data-Level Exclusion Criteria**

1. Incomplete measurement sets (missing amplitude, latency, or velocity data)
2. Technical failures during recording
3. Corrupted or inaccessible digital files
4. Non-standard recording protocols

**3.3 Interventions**

**3.3.1 AI Algorithm Version**

The study will use INSPIRE version 1.0, a multi-agent framework built upon "ChatGPT-4o" LLMs hosted within the AZURE AI platform, using the AutoGen 0.4 library. The system utilizes a bidirectional sequential multi-agent architecture with SelectorGroupChat functionality, enabling dynamic, context-aware collaboration between AI agents.

**Technical Validation Metrics**

Prior to the trial, the model achieved the following performance metrics during retrospective analysis:

- accuracy of 92.2% for detecting normal versus abnormal tests
- Native Gemini 1.5-pro achived 62.6% accuracy for detecting normal versus abnormal tests
- INSPIRE demonstrated significantly higher AIGERS scores overall and across the domains of finding and clinical diagnosis, (p<0.001) in contrast to Gemini 1.5-pro.

**3.3.2 Input Data Acquisition and Selection**

The input data for the AI intervention will consist of:

1. Patient demographic information (age, sex, clinical indication)
2. Medical history relevant to neuromuscular outpatient clinic
3. Complete nerve conduction study data (tables of measurements)
4. Needle EMG recordings (quantitative and qualitative descriptions)
5. Visual representations of waveforms

**3.3.3 Handling Poor-Quality Input Data**

If the EDX data contains artifacts or is of insufficient quality, the following procedure will be implemented:

1. If specific measurements are missing but the overall study is interpretable, the report will be discarded base upon the examiner decision
2. If the data quality falls below a predetermined threshold (>30% of measurements affected by artifacts or >2 critical measurements missing), the case will be excluded from AI analysis and documented as a technical failure

For both arms, poor-quality data will be documented.

**3.3.4 Human-AI Interaction**

The human-AI interaction in handling input data involves:

1. The EDX technician and/or physician performing the studies according to standard protocols
2. A research coordinator verifying data completeness and transferring it to the AI system
3. The AI system generating a preliminary report
4. The physician reviewing the AI-generated report alongside the raw data

**3.3.5 AI System Output**

The output will be formatted as a structured text document with sections clearly delineated, similar to conventional EDX reports but with standardized terminology and organization.

**3.4 Outcomes**

**3.4.1 Primary Outcome**

The primary outcome is the quality of EDX reports as measured by the AI-Generated EMG Report Score (AIGERS). This composite score (range 0-1) evaluates report quality based on:

1. Finding Score (50%): Accuracy and completeness of reported EDX findings
2. Clinical Diagnosis Score (50%): Accuracy and appropriateness of the clinical interpretation

The AIGERS score will be independently assessed by three board-certified neurologists who are not involved in the report generation and are blinded to the study arm.

**3.4.2 Secondary Outcomes**

1. Word count and comprehensiveness of reports
2. Physician satisfaction with the AI tool (5-point Likert scale survey)
3. Number of recommendations included in reports
4. Technical performance metrics for the AI system (sensitivity, specificity for detecting abnormal studies)
5. Inter-rater reliability of reports assessed by kappa scores

**3.5 Participant Timeline**

The study will follow a simple timeline:

1. Screening and enrollment prior to scheduled EDX appointment
2. Randomization on the day of the EDX study
3. EDX testing performed according to standard clinical protocols
4. Report generation (either AI-assisted or physician-only)
5. Quality assessment and data collection
6. No follow-up visits required for the study (though patients may have clinical follow-up as determined by their treating physician)

**3.6 Sample Size**

A trial with a sample size of 200 participants (100 per treatment group) will be conducted to rapidly assess outcomes. Should the results prove favorable, there is potential for expanding the sample size and the trial itself.

**3.7 Recruitment**

Participants will be recruited from patients referred for routine EDX testing at the neuromuscular clinic. All eligible patients will be approached for participation during a four-week period (January 21 to February 21, 2025). Based on clinic volume, we anticipate being able to enroll the required 200 participants within this timeframe.

**4. Methods: Assignment of Interventions**

**4.1 Allocation**

**4.1.1 Sequence Generation**

Participants will be randomized using a computer-generated random number sequence with a 1:1 allocation ratio.

**4.1.2 Concealment Mechanism**

Allocation will be concealed using sequentially numbered, opaque, sealed envelopes that will be opened only after the participant has been enrolled and immediately before the EDX study begins.

**4.1.3 Implementation**

The allocation sequence will be generated by a biostatistician not involved in patient enrollment. Research coordinators will enroll participants, and the treating neurologists will assign participants to interventions based on the randomization envelope.

**4.2 Blinding**

Due to the nature of the intervention, the neurologists generating the reports cannot be blinded to the study arm. However, the three neurologists assessing the quality of the reports (outcome assessors) will be blinded to the method of report generation. Reports from both arms will be reformatted to a standard template to prevent identification of the study arm based on formatting or style.

**5. Methods: Data Collection, Management, and Analysis**

**5.1 Data Collection Methods**

**5.1.1 Data Collection Instruments**

1. EDX raw data: Collected using standard EDX equipment (Viking or Synergy systems)
2. AIGERS assessment forms: Standardized forms for blinded reviewers to score report quality
3. Physician satisfaction surveys: Electronic surveys using REDCap
4. Time tracking software: To measure report generation time
5. AI system logs: To capture technical performance metrics

**5.1.2 Data Management**

All data will be stored on secure, password-protected servers with encryption. Data entry will be performed by trained research coordinators with regular quality checks. The database will include validation rules to minimize entry errors.

**5.1.3 Data Security for AI Systems**

The following specific security measures will be implemented for the AI system:

1. All API calls to the Azure AI platform will use encrypted HTTPS connections
2. Authentication will use Azure Active Directory with multi-factor authentication
3. Access to the AI system will be restricted to authorized users with role-based permissions
4. All system interactions will be logged with user identification, timestamp, and action details
5. Patient data will be de-identified before processing by the AI system
6. Data at rest will be encrypted using AES-256 encryption
7. Regular security audits will be performed to identify vulnerabilities
8. All system components will comply with HIPAA and local data protection regulations

### 5.2 Statistical Methods

#### 5.2.1 Sample Size

The study will enroll 200 participants (100 per arm) during the one-month study period at the neuromuscular outpatient clinic at Rambam Healthcare Campus. While we acknowledge that this sample size may be insufficient to detect small differences between groups, it will provide initial insights into the performance differences between AI-assisted and physician-only approaches and inform future larger studies if results suggest potential benefits.

#### 5.2.2 Primary Outcome Analysis

The primary analysis will compare the AIGERS scores between the AI-assisted physician group and the physician-only group. We will first check for normality using the Shapiro-Wilk test. If data are not normally distributed, which is anticipated based on similar metrics, we will use the non-parametric Kruskal-Wallis test for the primary comparison. A two-sided p-value <0.05 will be considered statistically significant.

In addition to the main comparison between intervention and control groups, we will also evaluate the AI-alone performance by comparing the AI-generated reports (before physician modification) with both the AI-assisted physician and physician-only groups using the same statistical approach.

#### 5.2.3 Secondary Outcome Analyses

For secondary analyses, the following statistical methods will be used:

1. **Continuous Variables**: Analyzed using Kruskal-Wallis tests (due to anticipated non-normal distributions) for three-way comparisons among AI-alone, AI-assisted, and physician-only groups. For direct comparison between AI-assisted and physician-only groups, Mann-Whitney U tests will be used.
2. **Categorical Variables**: Compared using chi-square or Fisher's exact tests as appropriate based on expected cell counts.
3. **Interrater Reliability**: Assessed using Fleiss' kappa for multiple raters (for the three independent evaluators scoring report quality) and Cohen's kappa for assessing agreement between AI and AI-Physician groups.
4. **Report Characteristics**:
   - Word count will be compared between groups using non-parametric tests
   - Number of recommendations included in reports will be compared between groups
   - Number of non-relevant recommendations will be quantified and compared
5. **Physician Satisfaction**: Analyzed using descriptive statistics for Likert scale responses (mean, standard deviation) and thematic analysis for free-text comments.

#### 5.2.4 Data Distribution and Transformation

Based on prior experience with similar metrics, we anticipate that the AIGERS scores and other continuous measurements may not be normally distributed. We will apply appropriate transformations if necessary to achieve normality. If transformations are unsuccessful, non-parametric tests will be used as the primary analytical approach.

#### 5.2.5 Handling of Missing Data

Missing data will be addressed as follows:

1. Cases where EDX data quality is insufficient for AI analysis will be documented as technical failures
2. For the primary outcome analysis, we will use complete case analysis if missing data is <5%
3. If missing data exceeds 5%, multiple imputation techniques will be employed
4. Sensitivity analyses will be conducted to assess the impact of missing data on outcomes

#### 5.2.6 Subgroup and Exploratory Analyses

1. **Diagnostic Category Subgroups**: Separate analyses for common diagnostic categories (radiculopathy, mononeuropathy, polyneuropathy) if sufficient numbers are available in each subgroup.
2. **Normal vs. Abnormal Studies**: Stratified analysis comparing AI performance on normal versus abnormal EDX studies.
3. **Physician Experience**: Exploratory analysis of whether physician experience level modifies the effect of AI assistance.
4. **Error Pattern Analysis**: Detailed categorization and analysis of error types made by AI versus physicians.

All secondary and exploratory analyses will be clearly identified as such in reporting, and appropriate corrections for multiple testing will be applied when interpreting results.

**6. Methods: Monitoring**

**6.1 Data Monitoring**

Given the short duration and minimal risk nature of the study, a formal data monitoring committee is not required. Regular monitoring will be conducted by the steering committee.

**6.2 Harms**

Adverse events related to the EDX procedure itself will be recorded and managed according to standard clinical protocols. The study adds no additional physical risk to patients.

**6.3 Performance Error Analysis**

The study will systematically identify and analyze AI performance errors, defined as:

1. Misclassification of normal vs. abnormal studies
2. Incorrect interpretation of specific findings
3. Inappropriate diagnostic conclusions
4. Missing critical information
5. Technical failures of the AI system

For each error, we will document:

- Type and severity of error
- Potential clinical impact
- Whether the error was detected and corrected by the reviewing physician
- Root cause analysis (technical limitation, data quality issue, etc.)
- Potential solutions or improvements

A dedicated log of performance errors will be maintained throughout the trial and will inform future system improvements. Regular reviews of error patterns will be conducted by the technical team and clinical experts.

**7. Ethics and Dissemination**

**7.1 Research Ethics Approval**

This study has been approved by the Institutional Review Board of Rambam Healthcare Campus.

**7.2 Protocol Amendments**

Any modifications to the protocol will be communicated to the IRB, trial registry, and all study team members. Significant amendments will require IRB approval before implementation.

**7.3 Consent**

Written informed consent will be obtained from all participants by trained research staff prior to enrollment. Participants will be informed that their EDX studies will be used to test an AI system but that their clinical care will not be affected.

**7.4 Confidentiality**

All participant information will be de-identified for analysis. Data will be stored on secure servers with access limited to study personnel. All team members will complete training in human subjects protection and data security.

**7.5 Declaration of Interests**

All investigators have reported no conflicts of interest related to this study.

**7.6 Access to Data**

The final trial dataset will be accessible to the principal investigators and the data analysis team. Other researchers may request access to de-identified data after the primary analyses are complete.

**7.7 AI Intervention Access**

The INSPIRE AI framework code and model architecture will be made available for research purposes upon request after study completion, subject to intellectual property considerations. Documentation of the system architecture, training methodology, and limitations will be provided alongside any shared code. Access to the actual trained model may be limited by licensing restrictions from the underlying LLM provider (Microsoft Azure).

**7.8 Dissemination Policy**

Study results will be published in peer-reviewed journals regardless of outcome and presented at scientific conferences. Participants may request a summary of the findings. All publications will follow CONSORT-AI reporting guidelines.
